# Effect of Botulinum Toxin on Non-Motor Symptoms in Cervical Dystonia

**DOI:** 10.3390/toxins13090647

**Published:** 2021-09-12

**Authors:** Matteo Costanzo, Daniele Belvisi, Isabella Berardelli, Annalisa Maraone, Viola Baione, Gina Ferrazzano, Carolina Cutrona, Giorgio Leodori, Massimo Pasquini, Antonella Conte, Giovanni Fabbrini, Giovanni Defazio, Alfredo Berardelli

**Affiliations:** 1 Department of Human Neurosciences, Sapienza University of Rome, Viale dell’Università 30, 00185 Rome, Italy; matteo.costanzo@uniroma1.it (M.C.); daniele.belvisi@uniroma1.it (D.B.); annalisa.maraone@uniroma1.it (A.M.); viola.baione@uniroma1.it (V.B.); gina.ferrazzano@uniroma1.it (G.F.); carolina.cutrona@uniroma1.it (C.C.); giorgio.leodori@uniroma1.it (G.L.); massimo.pasquini@uniroma1.it (M.P.); antonella.conte@uniroma1.it (A.C.); giovanni.fabbrini@uniroma1.it (G.F.); 2 IRCSS Neuromed, Via Atinense 18, 86077 Pozzilli, Italy; 3 Department of Neurosciences, Mental Health and Sensory Organs, Faculty of Medicine and Psychology, Suicide Prevention Centre, Sant’Andrea Hospital, Sapienza University of Rome, Via di Grottarossa 1035-1039, 00185 Rome, Italy; isabella.berardelli@uniroma1.it; 4 Department of Medical Sciences and Public Health, University of Cagliari, SS 554 Bivio Sestu, 09042 Monserrato, Italy; giovanni.defazio@unica.it

**Keywords:** cervical dystonia, non-motor symptoms, botulinum toxin, psychiatric disorders, pain

## Abstract

Patients with cervical dystonia (CD) may display non-motor symptoms, including psychiatric disturbances, pain, and sleep disorders. Intramuscular injection of botulinum toxin type A (BoNT-A) is the most efficacious treatment for motor symptoms in CD, but little is known about its effects on non-motor manifestations. The aim of the present study was to longitudinally assess BoNT-A’s effects on CD non-motor symptoms and to investigate the relationship between BoNT-A-induced motor and non-motor changes. Forty-five patients with CD participated in the study. Patients underwent a clinical assessment that included the administration of standardized clinical scales assessing dystonic symptoms, psychiatric disturbances, pain, sleep disturbances, and disability. Clinical assessment was performed before and one and three months after BoNT-A injection. BoNT-A induced a significant improvement in dystonic symptoms, as well as in psychiatric disturbances, pain, and disability. Conversely, sleep disorders were unaffected by BoNT-A treatment. Motor and non-motor BoNT-A-induced changes showed a similar time course, but motor improvement did not correlate with non-motor changes after BoNT-A. Non-motor symptom changes after BoNT-A treatment are a complex phenomenon and are at least partially independent from motor symptom improvement.

## 1. Introduction

Cervical dystonia (CD), the most common form of idiopathic focal dystonia [1,2], is characterized by involuntary muscle contractions of the cervical region causing abnormal movements and postures of the neck and head and tremor [3,4,5,6,7,8].

Although CD has long been considered mainly a motor disorder, in recent decades, increased attention has been focused on the possible presence of non-motor symptoms (NMS) throughout the disease course. In CD, the most frequent NMS are psychiatric symptoms [9,10,11,12,13], as well as sleep habit changes [14,15,16] and pain [17,18]. Growing evidence suggests that NMS play a pivotal role as determinants of quality of life and disability in CD patients [19,20,21], and a recent cross-sectional study has demonstrated that NMS might also intervene in determining clinical heterogeneity [22]. Finally, the relationship between motor and NMS is still a matter of debate. Most studies have reported that NMS are independent of CD motor manifestations [13,20,22], but it has also been suggested that NMS may represent a secondary manifestation of motor burden [23]. 

Intramuscular injection of botulinum toxin type A (BoNT-A) is currently considered the treatment of choice for CD since it is well-tolerated and effective in determining dystonia improvement [24,25]. BoNT-A is known to inhibit acetylcholine release from neuromuscular junctions, resulting in biochemical denervation of the treated muscle [26,27]. In addition to a primary peripheral clinical site of action, evidence also suggests that pharmacological properties of BoNT-A include central effects [27,28,29], including the modulation of glutamate, noradrenaline, dopamine, and glycine transmission and changes in the electrophysiologic and morphologic properties of central neurons [30]. A few studies have investigated the effect of BoNT-A on NMS in CD, though they focused on only one non-motor domain [31,32]. 

In the present study, we aimed to investigate the possible effect of BoNT-A treatment on non-motor burden, including psychiatric symptoms, pain, and sleep disorders, in CD patients. A further aim was to evaluate the relationship between BoNT-A-induced changes in motor manifestations and the effect of BoNT-A on NMS. For these purposes, we performed a longitudinal evaluation in a cohort of 45 CD patients. We tested motor and NMS at three different timepoints—before BoNT-A injection and one and three months after BoNT-A treatment. 

## 2. Results

Demographic and clinical features of patients with CD are reported in Table 1.

### 2.1. BoNT-A Treatment Parameters

Our cohort included 43 patients who were already on treatment with BoNT-A and 2 de novo patients. The mean duration of treatment was 10.5 ± 10.7 years and the number of treatment cycles was 38.6 ± 39. At baseline evaluation, 21 subjects received aboBoNT- A, 18 subjects received onaBoNT-A, and 6 subjects received incoBoNT-A. The mean total dose of abobotulinumtoxinA was 445.8 ± 192.2 units, and the mean total doses of incobotulinumtoxinA and AonabotulinumtoxinA were 90 ± 25.3 units and 121.8 ± 35.2 units, respectively. The most frequently injected muscle groups at baseline were the splenius capitis, sternocleidomastoid, trapezius, and levator scapulae. No EMG or ultrasound assessment was performed. 

### 2.2. Effects of BoNT-A on Motor and Non-Motor Symptoms in Patients with CD

**Figure 1:**
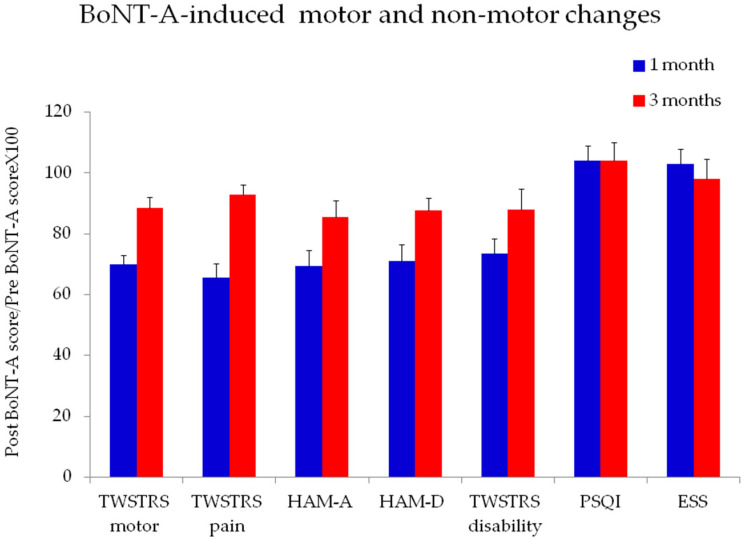
BoNT-A-induced changes at one- and three-month evaluations in motor and non-motor symptoms in CD patients. Error bars denote standard error. (TWSTRS: Toronto Western Spasmodic Torticollis Rating Scale; HAM-A: Hamilton Anxiety Rating Scale; HAM-D: Hamilton Depression Rating Scale; PSQI: Pittsburg sleep quality index; ESS: Epworth Sleepiness Scale).

**Figure 2:**
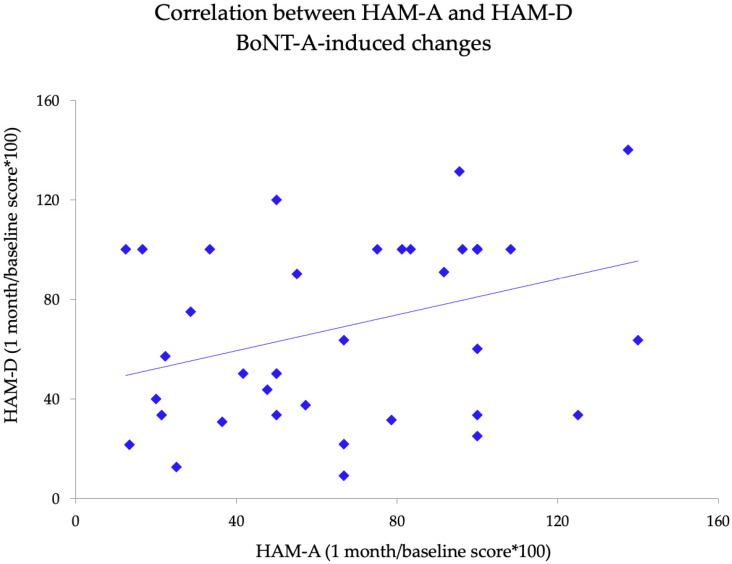
Correlation between BoNT-A-induced changes on Hamilton anxiety rating scale (expressed as the “HAM-A total score at one month/baseline total score *100”) and Hamilton depression rating scale scores (expressed as the “HAM-D total score at 1 month/baseline total score *100”).

The one-month follow-up evaluation showed that BoNT-A treatment induced a significant improvement in motor symptom severity, as shown by the significant reduction in TWSTRS motor section score (Z = −5.46; *p* < 0.00001) (Figure 1). Similarly, the TWSTRS pain section score was significantly lower one month after BoNT-A injection (Z = −4.91; *p* < 0.00001) (Figure 1). Regarding psychiatric disturbances, we observed that BoNT-A significantly reduced HAM-A (Z = −3.86; *p* = 0.0001) and HAM-D scores (Z = −3.1; *p* = 0.001) (Figure 2) but left the TWSTRS psychiatric section score unchanged (Z = −1.75; *p*= 0.07). Four of the 45 patients included in the study had a known diagnosis of depression and were on antidepressant drugs. BoNT-A did not modify sleep disorders, as tested by PSQI score (Z = −1.85; *p* = 0.06), or excessive daily sleepiness, as tested by ESS score (Z = −0.13; *p* = 0.8). The TWSTRS dystonia-related disability score significantly improved after BoNT-A (Z = −3.91; *p* < 0.0001), but global perceived disability, as tested by IPDS score, was unchanged after BoNT-A (Z = −0.05; *p*= 0.95). At the three month follow up evaluation, we observed a slighter but still significant improvement in TWSTRS motor section (Z = −3.59; *p* = 0.0003), TWSTRS pain section (Z = −3.94; *p* = 0.0001), HAM A (Z = −3.28; *p* = 0.001) and HAM-D scores (Z = −3-05; *p* = 0.002) (Figure 1). 

### 2.3. Relationship between Motor and Non-Motor BoNT-A-Induced Changes 

We analyzed the possible correlations between clinical variables that were significantly modified by BoNT-A treatment. These included TWSTRS motor section, TWSTRS pain section, HAM A and HAM-D scores. Spearman’s correlation coefficient showed no significant correlations between motor and non-motor BoNT-A-induced changes (all expressed as the ratio between one month and baseline clinical scale scores) (all *p* > 0.05). Conversely, we observed a significant correlation between HAM-A and HAM-D score changes one month after BoNT-A (r = 0.48; *p* = 0.001) (Figure 2). In addition, we did not observe any correlation between psychiatric and pain scores (all *p* > 0.05). 

**Figure 3:**
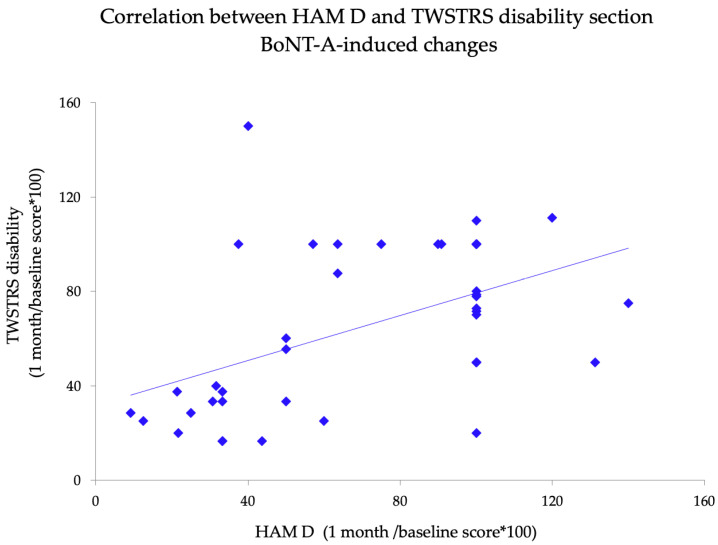
Correlation between BoNT-A-induced changes on Hamilton Depression Rating Scale scores (expressed as the “HAM-D total score at one month/baseline total score *100”) and TWSTRS dystonia-related disability score (expressed as the “TWSTRS disability total score at one month/baseline total score *100”).

Given that TWSTRS dystonia-related disability scores were also significantly changed by BoNT-A, we investigated possible correlations between this variable and motor and non-motor variables. We observed that disability BoNT-A-induced changes did not correlate with motor changes but significantly correlated with HAM-D score after BoNT-A changes (r = 0.38; *p* = 0.01) (Figure 3). No significant correlations between pain score and other non-motor symptoms scores were found (all *p* > 0.05). Similarly, we did not observe any significant correlation between site of injection and BoNT-A-induced effects on motor and non-motor symptoms.

## 3. Discussion

In the present paper, we longitudinally evaluated the effect of BoNT-A intramuscular injection on motor and non-motor manifestations in a cohort of 45 CD patients. Beyond the expected improvement in motor symptom severity and dystonia-related disability, we observed that BoNT-A intramuscular injection improved pain and psychiatric disturbances but not sleep disorders. We did not observe any association between motor and non-motor symptom improvements after BoNT-A treatment. Finally, we found that the reduction in dystonia-related disability after BoNT-A treatment was correlated with psychiatric disturbance changes but not with motor symptom severity reduction.

To exclude methodological biases that could have affected our results, we took several precautions. To minimize selection bias, we consecutively recruited CD patients who were diagnosed according to international clinical diagnostic recommendations in a single center setting [33,34], thus providing a case series resembling the general population. To exclude any confounding effects due to previous BoNT injections, a baseline clinical assessment was performed at least four months after the last BoNT treatment. Finally, to avoid observer bias, baseline and follow-up clinical examinations were performed by a single neurologist expert in movement disorders who administered standardized scales to evaluate motor and NMS. Similarly, psychiatric evaluation was performed by a single psychiatrist expert in psychiatric conditions in patients with movement disorders. The possibility that a previous history of depression might have affected our results is highly unlikely since at the time of evaluation only four of 45 patients tested suffered from depression and were on antidepressant treatment.

In our study, we observed that BoNT-A injection was associated with a reduction in anxiety and depressive symptom severity, as indicated by the decrement in HAM-A and HAM-D scores, respectively. To our knowledge, no studies have specifically evaluated the effect of BoNT-A on psychiatric symptoms in CD. One study demonstrated an improvement in anxiety and depression following BoNT-A intramuscular injection in another frequent form of focal dystonia, blepharospam (BSP) [35]. Differently from our study, the authors did not investigate the extent of motor symptom improvement in BSP and therefore it is not possible to establish whether psychiatric improvement paralleled motor improvement or not. The most intuitive explanation to interpret BoNT-A-induced changes on psychiatric measures in CD is that the symptomatic improvement in motor impairment, dystonia-related disability, and pain contributes to reducing feelings of depression and anxiety in these patients. In line with this hypothesis, we observed a similar time course for motor and psychiatric symptom improvement after BoNT-A injection. This is also consistent with findings by Jahanshahi and Marsden, who hypothesized that the occurrence of depressive symptoms in CD is related to negative body image, secondary to abnormal postures of the head [36,37,38]. Contrary to the hypothesis that BoNT-A effects on psychiatric disturbances mainly depend on motor symptom improvement, we did not find any correlation between psychiatric and motor domain changes after BoNT-A injection. The mechanism linking motor and neuropsychiatric features is unclear, but it has been suggested that CD is a network disorder in which motor and NMS depend on the activity of independent but integrated nodes [22], including the limbic fronto-striatal circuitry, which connects the anterior cingulate and orbitofrontal cortex with ventral striatal areas, the ventral pallidum, and the medial thalamus [39,40,41]. Recent imaging studies have suggested that BoNT treatment determines a rebalancing of connectivity in the sensorimotor network in CD patients [41,42,43]. From this perspective, it is possible that improvement in psychiatric disturbances depends on a rebalance in connectivity between motor and non-motor structures involved in the CD brain network [44,45,46]. Future neuroimaging and neurophysiological studies investigating functional connectivity between brain network nodes underlying motor and psychiatric manifestations are, however, needed to confirm this hypothesis. 

In the present study, we also observed that BoNT-A treatment may temporarily relieve pain in CD patients, as shown by the significant reduction in TWSTRS pain section score at the one-month evaluation, with a gradual return to baseline values at the three-month assessment. The short- and long-term effects of BoNT-A in reducing pain in CD patients have been consistently reported in previous studies [32,47,48]. It has been hypothesized that BoNT-A reduces pain via muscular relaxation with a reduction in painful ischemia in hypercontracted muscles or, alternatively, via inhibition of neurogenic inflammation and peripheral sensitization (see Marciniec et al., 2019 for a review) [48]. The present observation that BoNT-A-induced motor improvement did not correlate with changes in pain intensity suggests that the antinociceptive effect and muscle relaxation mechanisms of BoNT-A could be distinct phenomena. 

In addition, it is known that psychiatric disorders and pain strongly interact in patients affected by chronic conditions [49]. It is therefore possible that the improvement in pain after BoNT-A treatment may partly depend on the improvement of anxiety and depression observed in our patients and vice versa. Against this hypothesis we did not observe any correlation between pain and psychiatric symptom improvement after BoNT-A treatment. This suggests that BoNT-A induces a NMS improvement in CD throughout mechanisms that are at least partially distinct and symptom-specific. 

Unlike what was observed for psychiatric disorders and pain, we did not observe any effect of BoNT-A treatment on sleep quality and daytime sleepiness. Sleep habit disturbances represent conditions that are usually persistent or long-lasting and that are due to several variables, including environmental factors, occupational factors, physiologic changes, medical disorders, and psychiatric disorders. Due to the chronic nature of the condition and to the large number of variables that influence this non-motor symptom, it is possible that the short-term prospective design of our study did not allow us to obtain reliable results. Future longitudinal studies may shed new light on this topic in CD.

Similar to previous studies [25,50], we found that BoNT-A intramuscular injection determines a reduction in dystonia-related disability. We found that disability domain scores did not correlate with motor symptom severity, but significantly correlated with psychiatric domain scores. Most studies that have investigated the relationship between motor burden and non-motor features reported similar results, indicating that neuropsychiatric disturbances may play a role in disability in CD [22,51]. It is therefore plausible that disability in CD is a result of the contribution of motor and NMS, including pain and neuropsychiatric features. This finding highlights the importance of a multidisciplinary approach to dystonia management that uses treatment strategies aimed at reducing the severity of motor and non-motor domains. 

In order to assess NMS, we used a large number of clinical scales. It is important to point out that we obtained different results when we administered clinical scales assessing similar non-motor domains. For instance, we found that the TWSTRS psychiatric section score was unchanged following BoNT-A treatment, whereas HAM-A and HAM-D scores were significantly reduced. These contrasting results may be explained by the different clinimetric properties of these assessment scales in capturing various aspects of anxiety and depression. The TWSTRS psychiatric section is a screening tool used to rate several categories of psychiatric disturbance that are associated with CD and identify psychiatric disorders that may require further investigation and treatment [52,53]. HAM-A and HAM-D, in contrast, are assessment tools designed to provide a more complete picture and to rate the severity of general anxiety disorder and depression, respectively [54,55,56]. It is therefore possible that HAM-A and HAM-D might be more suitable than the TWSTRS in documenting the results of intervention programs, including pharmacotherapy or psychotherapy.

We acknowledge some limitations. The short-term prospective design of our study, with three evaluations (baseline and one and three months after treatment) did not allow us to observe BoNT-induced changes beyond this time. It is, however, likely that BoNT-A may determine a long-term effect on non-motor symptoms in CD. Therefore, future long-term studies simultaneously assessing BoNT-A’s effects on NMS will be necessary. Similarly, we did not include a two-month time point because one-month and three-month evaluations are more informative since they represent the peak and end phase of BoNT-A therapeutic effects, respectively. The use of only the BoNT-A type of botulinum toxin does not allow us to generalize the conclusions of this study to other types of botulinum toxins. Future studies directly comparing the effects of BoNT-A and BoNT-B treatment on non-motor symptoms in CD are warranted to clarify this aspect. Finally, we did not include a placebo-treated group for ethical reasons, and we cannot fully exclude a repetition bias during the administration of clinical scales. It is, however, important to point that after BoNT-A treatment we observed a similar time course between motor symptom severity, which was directly evaluated by the examinator, and NMS severity, which was assessed by the administration of clinical scales. 

## 4. Conclusions

In conclusion, our findings suggest that BoNT-A intramuscular injection in CD patients is able to improve non-motor variables (psychiatric symptoms and pain) in patients affected by CD. Non-motor and motor symptom improvement after BoNT-A injection show a similar time course. BoNT-A effect on CD NMS may reflect a global improvement secondary to dystonic symptom reduction. On the other hand, the lack of correlation between motor and non-motor manifestation improvement after BoNT-A suggests that other non-motor domain-specific mechanisms intervene in determining non-motor improvement in CD. Our study underscores the importance of the detection and treatment of NMS in the management of CD patients and sheds light on the possible therapeutic role of BoNT-A on NMS in patients affected by CD.

## 5. Materials and Methods

### 5.1. Study Participants and Ethics

Forty-five patients with primary CD were consecutively enrolled from among outpatients at the movement disorder clinic of the Department of Human Neurosciences, Sapienza University of Rome (Table 1). Patients were included in the study if they were diagnosed with idiopathic CD according to validated criteria [1,33,34]. Exclusion criteria were a known genetic form of dystonia, secondary forms of CD, other neurological disorders, a history of exposure to drugs known to negatively impact cognitive and behavioral functions, and previous or current alcohol abuse. Demographic and clinical data, including gender, age, age at disease onset, disease duration, and BoNT-A dose, were collected. The experimental procedure was approved by the local institutional review board (Sapienza University of Rome Ethics Committee, No. 5941) and was conducted in accordance with the Declaration of Helsinki. All patients were informed of the purpose of the study and written informed consent was obtained from all participants.

### 5.2. Motor and Non-Motor Assessment 

All CD patients underwent three evaluations of motor symptoms: before (baseline) and one and three months after BoNT-A treatment.

The baseline assessment was performed 16 weeks after the last BoNT-A injection and immediately before a new BoNT-A treatment. The one-month evaluation was chosen because this time point corresponds to the peak effect of BoNT-A induced motor improvement [25,57,58,59]. The three-month evaluation was chosen to assess whether BoNT-A-induced changes in motor and non-motor symptoms show a similar temporal trajectory. Indeed, it is known that BoNT-A’s effects wear off at 16 weeks but they are still present, to a small extent, after 12 weeks [57,58,59]. 

A full neurological examination was performed by a neurologist expert in movement disorders. CD severity was assessed according to the revised Toronto Western Spasmodic Torticollis Rating Scale (TWSTRS) [52]. The scale includes 21 items divided into four sections. The first evaluates the amplitude of excursion of abnormal neck posture without opposing any movement while performing tasks indicated by the examiner. The second section evaluates the degree of disability of patients and their ability to work or perform housework, perform daily living activities (such as feeding, dressing, washing, shaving, etc.), drive, read, or watch television, or perform other activities outside the house (such as shopping, going to the movies, dining, or other recreational activities). The third section assesses severity and duration of pain and its contribution to patient disability. The fourth section evaluates the presence of psychiatric symptoms in the month preceding the evaluation. It is composed of six items and evaluates the presence of depressed feelings, loss of interest in things the patient used to enjoy, fear of performing activities (such as speaking, eating, or writing) in front of other people, feelings of anxiety, presence of panic attacks, and fear of going out of the house alone, being in crowds, standing in line, or traveling on buses or trains. To better evaluate motor symptom severity, all patients also underwent a standardized video recording [22,60,61,62].

NMS severity of CD patients was also assessed at three different time-points: before and one and three months after BoNT-A. The Hamilton Anxiety Rating Scale (HAM-A) was used to evaluate the presence and severity of anxiety [55], whereas the Hamilton Depression Rating Scale (HAM-D) was used to assess the presence and severity of depression [56]. To evaluate pain, we used the TWSTRS, section III, which evaluates the severity and duration of pain and its contribution to disability [52]. The presence of sleep disturbance was evaluated using the Pittsburgh Sleep Quality Index (PSQI) and the Epworth Sleepiness Scale (ESS) [63,64]. The PSQI is a self-rated questionnaire composed of 19 questions that assess sleep quality and disturbances in the month preceding its administration. The ESS is a self-administered questionnaire to measure daytime sleepiness. 

Finally, we investigated dystonia-related and global disability. Global disability was evaluated by administering the Italian Perceived Disability Scale (IPDS), a 20-item self-report instrument that assesses global disability on a five-point Likert scale (completely false to completely true) [65]. The IPDS is based on items that investigate people’s beliefs regarding autonomy/disability in different situations in life and has previously been used to assess perceived disability in patients affected by movement disorders [66,67].

### 5.3. Statistical Analysis

Data are expressed as mean ± standard deviation (SD) unless otherwise specified. Statistical analysis was performed by using IBM SPSS Statistics for Windows, Version 25.0. Armonk, NY, USA: IBM Corp. 25. The Shapiro–Wilk test was used to evaluate whether distribution was Gaussian or not. Parametric and non-parametric tests were used to compare clinical scale scores before and one and three months after BoNT-A, accordingly. Spearman’s rank correlation coefficient was used to evaluate possible correlations between BoNT-A-induced changes in motor and non-motor domains. For each patient, we calculated the ratio between one month and baseline clinical scale scores. In addition, we investigated possible correlations between disability and clinical scales by calculating the ratio between one month and baseline TWSTRS dystonia-related disability scores. A value of *p* < 0.05 indicated statistical significance. FDR correction was used to correct for multiple comparisons where appropriate.

## Figures and Tables

**Table 1:** Demographic and clinical features of patients with CD at baseline.

**Demographic and Clinical Data**	**CD Patients**
Females (%)	62%
Age in years (mean ± SD)	58.5 ± 12.8
Disease duration in years (mean ± SD)	10 ± 9.2
Schooling (years ± SD)	12.3 ± 3.5
Right-handed (%)	97%
Motor domain	
Tremor (%)	31%
TWSTRS severity Section ^1^ (mean ± SD)	11.5 ± 3.4
**Psychiatric domain**	
TWSTRS psychiatric Section ^1^ (mean ± SD)	5 ± 4.2
HAM-A ^2^ (mean ± SD)	9 ± 7.8
HAM-D ^3^ (mean ± SD)	8.3 ± 7.2
**Sleep domain**	
PSQI ^4^ (mean ± SD)	5.1 ± 3.7
ESS ^5^(mean ± SD)	4.1 ± 4.1
**Pain domain**	
TWSTRS pain Section ^1^ (mean ± SD)	14.6 ± 10.2
**Disability domain**	
TWSTRS disability Section ^1^ (mean ± SD)	6.8 ± 5.5
IPDS ^6^ (mean ± SD)	31.5 ± 16.7

^1^ TWSTRS: Toronto Western Spasmodic Torticollis Rating Scale. ^2^ HAM-A: Hamilton Anxiety Rating Scale. ^3^ HAM-D: Hamilton Depression Rating Scale. ^4^ PSQI: Pittsburgh Sleep Quality Index. ^5^ ESS: Epworth Sleepiness Scale. ^6^ IPDS: Italian Perceived Disability Scale.

## Data Availability

Data are available upon reasonable request.
